# Brighter Fluorescent Derivatives of UTI89 Utilizing a Monomeric vGFP

**DOI:** 10.3390/pathogens5010003

**Published:** 2016-01-05

**Authors:** Majid Eshaghi, Kurosh S. Mehershahi, Swaine L. Chen

**Affiliations:** 1 Department of Medicine, Yong Loo Lin School of Medicine, National University of Singapore, 1E Kent Ridge Road, NUHS Tower Block, Level 10, Singapore 119074, Singapore; eshaghim@gis.a-star.edu.sg (M.E.); mehershahiks@gis.a-star.edu.sg (K.M.); 2 Infectious Diseases Group, Genome Institute of Singapore, 60 Biopolis street, Genome, #02-01, Singapore 138672, Singapore

**Keywords:** GFP, uropathogenic *Escherichia coli*, urinary tract infection

## Abstract

Fluorescent proteins, especially green fluorescent protein (GFP), have been instrumental in understanding urinary tract infection pathogenesis by uropathogenic *Escherichia coli* (UPEC). We have used a recently developed GFP variant, vsfGFP-9, to create new plasmid- and chromosome-based GFP derivatives of the UPEC strain UTI89. The vsfGFP-9 strains are nearly 10× brighter with no *in vitro* growth or *in vivo* virulence defects compared to previously reported GFP-expressing UTI89 strains. The chromosomal vsfGFP-9 strain is equivalent to the wild type UTI89 during *in vivo* UTI, while both plasmid GFP constructs have an equivalent virulence defect compared to non-plasmid carrying UTI89. These new vsfGFP-9 expressing strains should be useful for further studies of the pathogenesis of UTI89, and similar strategies can be used to create improved fluorescent derivatives of other UPEC strains.

## 1. Introduction

Fluorescent proteins (FPs) have been instrumental to our understanding of bacterial pathogenesis [1]. FPs have been used widely in plasmid- or chromosome-based strategies in *in vitro* and *in vivo* studies [1,2]. Plasmid-based GFPs generally have higher copy number and expression, benefitting brightness at the cost of cell-to-cell variation (due to different copy numbers in different cells), plasmid instability, and fitness defects due to plasmid carriage or high GFP expression. Fitness defects in particular then complicate studies of pathogenesis, which more often manifest *in vivo*. In contrast, chromosomal GFPs tend to have lower expression and lower brightness, limiting utility for visualizing small (or individual) bacterial collections while mitigating these other problems with variability, stability, and fitness.

Urinary tract infections (UTIs) are one of the most common bacterial infections of humans, accounting for over $2.3 billion in medical expenditures annually in the US [3]. Most UTIs are caused by strains of *E. coli*, thus the term uropathogenic *E. coli* (UPEC). As with other infectious diseases [1,4], fluorescent proteins have been instrumental for many discoveries of the pathogenic mechanisms utilized by UPEC, including the development of intracellular bacterial communities (IBCs) [5,6], quiescent intracellular reservoirs (QIRs) [7], and avoidance of neutrophil killing [6] by the cystitis strain UTI89 [8]. For these studies, two strains are commonly used, both of which express the GFPmut3 variant of GFP [1]: UTI89 carrying plasmid pANT4 [9] and UTI89 *att_HK022_*::*COM-GFP* [10]. Both of these strains have been used to monitor formation of intracellular structures during UTI by microscopy [7,10,11], but to date UTI89/pANT4 has not been further characterized for other infection phenotypes.

Since the identification of GFPmut3, new variants of GFP demonstrate various improved properties [12]. One of these in particular, superfolder GFP (sfGFP) [13], has higher brightness and faster folding kinetics than the currently used GFPmut3. We have further improved the brightness of sfGFP by fusing it to a GFP-specific single domain antibody [14] using the vGFP strategy to create a monomeric fluorophore with 30%–50% increased brightness and pH resistance [15]. We refer to this improved sfGFP as vsfGFP-9.

We here report the creation of new derivatives of UTI89 carrying vsfGFP-9 on the chromosome or on a derivative of the pANT4 plasmid that provide nearly 10× increased brightness to the commonly used UTI89 *att_HK022_*::*COM-GFP* and UTI89/pANT4, respectively. We demonstrate that these derivatives, despite the markedly higher brightness, have no fitness defects relative to the strains they are intended to replace. Furthermore, we find that the plasmid-based strains (UTI89/pANT4 and SLC-638) have an equivalent fitness defect relative to UTI89 as measured by infection load. In contrast, chromosomal expression of vsfGFP-9 produces brightness approaching that of UTI89/pANT4 without a defect in IBC formation or infection load. These new, brighter strains should be useful in future studies of the pathogenic mechanisms of UTI89, and the strategies employed here can be similarly applied to improve fluorescent derivatives of other UPEC strains.

## 2. Results and Discussion

### 2.1. New vsfGFP-9 Expressing Derivatives of UTI89 Are 10× Brighter Than Former GFP Expressing Strains

**Figure 1:**
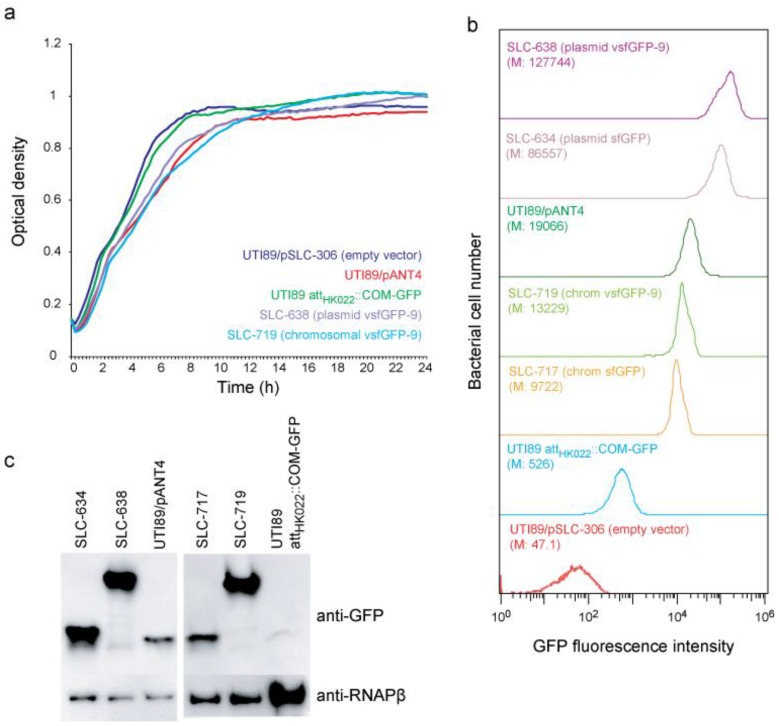
*In vitro* characterization of vsfGFP-9 derivatives of UTI89. (**a**) Growth curves in Lysogeny broth (LB) medium for the parental wt UTI89/pSLC-306 (empty vector control; dark blue), UTI89 *att_HK022_*::*COM-GFP* (green), SLC-719 (chromosomal vsfGFP-9; light blue), UTI89/pANT4 (red), and SLC-638 (plasmid vsfGFP-9; purple); (**b**) Flow cytometry analysis of green fluorescent protein (GFP) brightness for UTI89/pSLC-306 (red), UTI89 *att_HK022_*::*COM-GFP* (blue), SLC-717 (chromosomal sfGFP; brown), SLC-719 (light green), UTI89/pANT4 (dark green), SLC-634 (plasmid sfGFP; pink), and SLC-638 (purple). M indicates the median GFP fluorescence. (**c**) Quantification of GFP protein levels in UTI89 strains. (top) Immunoblot of samples from panel (**b**) using α-GFP antibody. α-RNAPβ was used as a loading control.

We generated UTI89 derivatives carrying plasmid (SLC-638) and chromosome (SLC-719) based vsfGFP-9 constructs intended to improve on UTI89/pANT4 and UTI89 *att_HK022_*::*COM-GFP*, respectively. As controls, we also created corresponding sfGFP derivatives (plasmid: SLC-634; chromosomal: SLC-717). The vsfGFP-9 strains had no gross growth defect relative to UTI89 or to their corresponding GFPmut3 strain (SLC-638 compared with UTI89/pANT4; SLC-719 compared with UTI89 *att_HK022_*::*COM-GFP*) (Figure 1a). By flow cytometry, we found that the strains carrying vsfGFP-9 were the brightest; both on the plasmid and on the chromosome, the vsfGFP-9 strains were nearly 10× brighter than their corresponding GFPmut3 strains and ~1.5× brighter than the corresponding sfGFP strain (Figure 1b). Based on Western blots for GFP, the increased brightness was due to both increased expression as well as the enhanced brightness of vsfGFP-9 over sfGFP and GFPmut3 (Figure 1c). Interestingly, SLC-719 (carrying a chromosomal vsfGFP-9) approached the brightness of UTI89/pANT4 (carrying a plasmid-based GFPmut3) (Figure 1b, light and dark green traces).

### 2.2. New Chromosomal vsfGFP-9 Construct Has No Fitness Defects during UTI Relative to Former GFP Expressing Strains

**Figure 2:**
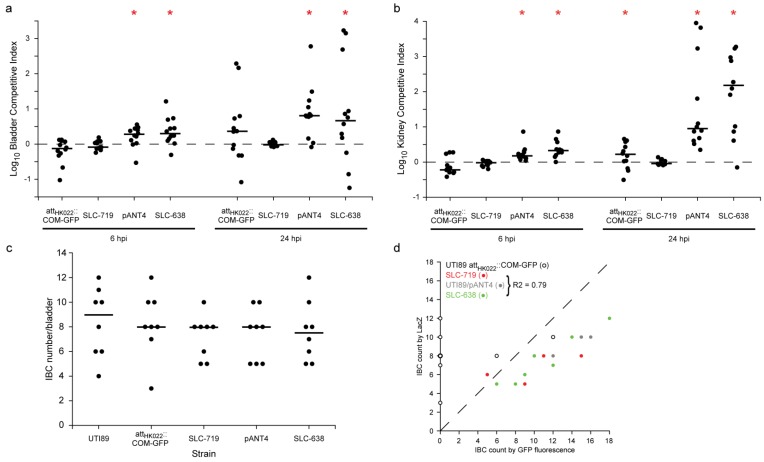
*In vivo* characterization of vsfGFP-9 derivatives of UTI89. Competitive infections between UTI89 *att_HK022_*::*COM-GFP*, SLC-719, UTI89/pANT4, and SLC-638 and the parental wt UTI89 were performed. (**a**) Logarithm of the competitive index against wt UTI89 in the bladder at 6 and 24 hpi. Higher values indicate wt UTI89 outcompetes the GFP strain; (**b**) Log competitive index against wt UTI89 in kidneys at 6 and 24 hpi. Red bars indicate median values. *, *p* < 0.05 (two-tailed Wilcoxon signed-rank test whether log competitive indices are different from 0). (**c**) Quantification of IBCs at 6 hpi; (**d**) Comparison of GFP *versus* LacZ staining to quantify IBCs for plasmid-based GFP expressing strains. Data from UTI89 *att_HK022_*::*COM-GFP* (open circles), SLC-719 (red), UTI89/pANT4 (gray), and SLC-638 (green) are shown. R^2^ coefficient for combined data from SLC-719, UTI89/pANT4, and SLC-638 is indicated in the legend.

Because plasmid carriage as well as high GFP expression can both lead to fitness defects *in vivo*, we tested the vsfGFP-9 constructs in an *in vivo* murine model of UTI. Using competitive infections against the parental (nonfluorescent and unmodified) UTI89, we generally saw no fitness defect at 6 hpi or 24 hpi for either UTI89 *att_HK022_*::*COM-GFP* or SLC-719 (chromosomal vsfGFP-9) in either the bladder or the kidney (Figure 2a,b); at 24 hpi in kidneys we saw a slight (<0.5 log) but significant defect in UTI89 *att_HK022_*::*COM-GFP*. In contrast, we saw a significant defect in competitive infections for both UTI89/pANT4 and SLC-638 (plasmid vsfGFP-9) relative to UTI89 at 6 hpi and 24 hpi; however, there was no significant difference in the competitive indices between these plasmid-carrying strains.

We next tested these strains for IBC formation. Validating previous reports that used GFPmut3 expressing strains to study IBCs, we found no significant difference in the number of IBCs formed by any of the strains tested relative to wt UTI89 as quantified by LacZ staining (Figure 2c). The fluorescent strains also enabled a more convenient quantification of IBCs by direct observation under a fluorescent dissecting microscope; again no significant difference was seen between UTI89/pANT4 and SLC-638, though the number of IBCs for each strain detected was slightly higher than (though well correlated with) LacZ staining (Figure 2d). Highlighting the brightness advantage of the new strains, among the chromosomal GFP expressing strains, we were only able to detect IBCs by fluorescence in SLC-719 but not with UTI89 *att_HK022_*::*COM-GFP* (except weakly in two cases; this was due to low fluorescence of this strain over background bladder fluorescence), which is consistent with the higher fluorescence of SLC-719 that we measured by FACS.

## 3. Experimental Section

*Strains and growth conditions*. UTI89 [8], UTI89 *att_HK022_*::*COM-GFP* [10], and UTI89/pANT4 [9,16] have been previously described. Lysogeny broth (LB) was used for all growth. Ampicillin was supplemented as required at 100 μg/mL, and kanamycin at 50μg/mL. For mouse infections, bacteria were grown under type 1 pili inducing conditions as previously described [17]. Bacterial growth curves were measured using a Bioscreen C MBR (Bioscreen, Finland) in LB medium at 37 °C for 24 h.

*Construction of sfGFP and vsfGFP-9 expressing strains*. All plasmids used in this study are listed in Table S1. All primers used for cloning and homologous recombination were purchased from Sigma (Singapore) and are listed in Table S2. The genes encoding sfGFP and vsfGFP-9 were amplified by PCR using primer pairs P1-P2 from plasmids pSLC-253 and pSLC-255 respectively. The PCR products were then digested and cloned into pANT4 (replacing GFPmut3) using *XbaI* and *HindIII* restriction sites to produce pSLC-282 (containing sfGFP) and pSLC-284 (containing vsfGFP-9). Nonfluorescent colonies from the pSLC-282 cloning contained plasmids without GFP, giving pSLC-306 (empty vector control for pANT4). pSLC-282 was transformed into UTI89 to give SLC-634; pSLC-284 was transformed into UTI89 to give SLC-638.

For chromosomal GFP strains, annealed complementary pairs of oligonucleotides (primer P3 and its reverse complement) containing a *rrnB*_T2 transcriptional terminator [18] upstream of a modified σ70 promoter [19] were digested with *ClaI* and *XbaI* and cloned into plasmids pSLC-253 and pSLC-255 digested with the same enzymes to generate pSLC-293 (sfGFP) and pSLC-294 (vsfGFP-9). We integrated sfGFP or vsfGFP-9 between chromosomal coordinates 1044461-62 in UTI89, which is the *att_HK022_* region, using a two-step positive-negative selection system [20]. Briefly, the kan-P*_rhaB_*-*relE* cassette was amplified from pSLC-217 [20] using primers P4 and P5 and integrated into the *att_HK022_* region using Red recombinase-mediated recombineering [21] to generate SLC-653. Primer pairs P5 and P6 were used to amplify fragments containing sfGFP or vsfGFP-9 from plasmids pSLC-293 or pSLC-294; these PCR products were used to replace the kan-P*_rhaB_*-*relE* cassette by Red recombinase-mediated recombineering with negative selection on M9 media supplemented with 2% rhamnose, giving SLC-717 (sfGFP) and SLC-719 (vsfGFP-9).

*Western blots*. Primary antibodies (and dilutions) used were anti-GFP mouse monoclonal antibody (1:6000; Santa Cruz Biotechnology, Shanghai, China), anti-RNA Polymerase β (1:6000; Santa Cruz Biotechnology, Shanghai, China). The secondary antibodies were ECL^™^ Anti-rabbit IgG and ECL^™^ Anti-mouse IgG, both conjugated with Horseradish peroxidase (HRP) and used at a 1:10,000 dilution.

*Mouse infections*. Infections were performed as previously described [17]. Data shown is the result of two separate experiments performed on separate days with four to six mice per strain and per time point. Six to seven-week old C3H/HeN female mice were obtained from Harlan (Israel). In co-infections with UTI89 and SLC-719 (chromosomal vsfGFP-9), SLC-719 colonies were differentiated from UTI89 cells by visualization of green fluorescence under 10× magnification because neither strain carries an antibiotic resistance cassette. In coinfections with UTI89 and UTI89 *att_HK022_*::*COM-GFP*, UTI89 *att_HK022_*::*COM-GFP* colonies were quantified by plating on LB supplemented with kanamycin, and UTI89 colonies were calculated by subtraction of UTI89 *att_HK022_*::*COM-GFP* titers from total titers quantified on LB plates. In each experiment, two to three of these LB plates were also used to quantify UTI89 *att_HK022_*::*COM-GFP* titers using green fluorescence under 10× magnification; in all cases, the fluorescence-based quantification was within 10% of the kanamycin-based quantification.

IBCs were quantified in single infections for UTI89, UTI89 *att_HK022_*::*COM-GFP*, UTI89/pANT4, SLC-717, SLC-719, and SLC-638. At 6 hpi, infected mice were sacrificed, and harvested bladders were hemisected, stretched onto silicone pads, and fixed with 3% paraformaldehyde (Sigma) as previously described [17]. IBCs from GFP-expressing strains were quantified by fluorescence at 10× magnification. All samples were then subjected to X-Gal staining for independent quantification as previously reported [11]. Data shown is the result of two separate experiments performed on separate days with four mice per strain per experiment.

*Flow cytometry*. Flow cytometry analysis for GFP expression was performed on a S3^™^ Cell Sorter (Bio-Rad, Hercules, CA, USA).

## 4. Conclusions

We have created new plasmid- and chromosome-based GFP expressing derivatives of UTI89. Through using a new vsfGFP-9 gene as well as optimization of expression, these are nearly 10× brighter than previously published GFP UTI89 derivatives, and have no *in vitro* or *in vivo* defects compared with previous GFP-expressing strains. These strains should be useful for future studies of UTI89 pathogenesis and for the creation of vsfGFP-9 derivatives of other UPEC and *E. coli*.

## Supplementary Materials

The following are available online at www.mdpi.com/ 2076-0817/5/1/3/s1, Table S1: Plasmids used in this work, Table S2: Primer sequences used in this study.
