# Supplementary material for: Brighter Fluorescent Derivatives of UTI89 Utilizing a Monomeric vGFP
Source: Pathogens. 2016 Jan 5;5(1):3. doi: 10.3390/pathogens5010003 (PMC4810124; doi:10.3390/pathogens5010003)
Supplement: Supplementary file 1 [file pathogens-05-00003-s001.pdf]

Supplementary Materials: Brighter Fluorescent Derivatives of UTI89 Utilizing a Monomeric vGFP

Majid Eshaghi, Kurosh S. Mehershahi and Swaine L. Chen

Table S1. Plasmids used in this work.

Plasmid	Description	Relevant Information
pBAD33	Plasmid carrying arabinose-inducible promoter	Lab stock
pSLC-253	pBAD33-sfGFP	Lab stock
pSLC-255	pBAD33-vsfGFP-9	Lab stock
pANT4	Carries GFPmut3 under P_{tac} [1]	Lab Stock
pSLC-282	pANT4 with GFPmut3 replaced by sfGFP	This study
pSLC-284	pANT4 with GFPmut3 replaced by vsfGFP-9	This study
pSLC-293	pBAD33 with Para replaced by$P_{\sigma70}$-sfGFP	This study
pSLC-294	pBAD33 with Para replaced by $P_{\sigma70}$-vsfGFP-9	This study
pSLC-306	pANT4 with GFPmut3 removed (empty vector)	This study

Table S2. Primer sequences used in this study.

Primer Name	Nucleotide Sequence (5′ to 3′)	Description
P1	TCTAGAATGAGCAAAGGAGAAGAACTTTTCACTGGA	pSLC-282 and pSLC-284 constructs
P2	CATCATCATCATCATCATTAA AAGCTT	pSLC-282 and pSLC-284 constructs
P3	ATCGATAGAAGGCCATCCTGACGGATGGCCTTTTATGCCATAGCATTTTTATCC TTGACGGCTAGCTCAGTCCTAGGTACAGTGCTAGCTCTAGA	pSLC-293 and pSLC-294 constructs
P4	GCGCCATCAGGGCAAAGCCCATCCAGAGTCTTCGGGTCAGGGTTAAATTCAC GGTCGGTGCGTGTAGGCTGGAGCTGCTTC	SLC-653
P5	ATAATAAGGCTTTATGCTAGATGCATTCTGCTTTGCGACT CAACCTTTTTCACCTAAAGTCATATGAATATCCTCCTTAG	SLC-653
P6	GCGCCATCAGGGCAAAGCCCATCCAGAGTCTTCGGGTCAGGGTTAAATTCAC GGTCGGTGCTTGACGGCTAGCTCAGTCCTA	SLC-717 and SLC-719
P7	ATAATAAGGCTTTATGCTAGATGCATTCTGCTTTGCGACTCAACCTTTTTCACC TAAAGTTTAATGATGATGATGATGATG	SLC-717 and SLC-719

References

1. Lee, A.K.; Falkow, S. Constitutive and inducible green fluorescent protein expression in *Bartonella henselae*. *Infect. Immun.* **1998**, *66*, 3964–3967.
